# Meditation in the Workplace: Does Mindfulness Reduce Bias and Increase Organisational Citizenship Behaviours?

**DOI:** 10.3389/fpsyg.2022.747983

**Published:** 2022-04-11

**Authors:** Emma Constance Williams, Vince Polito

**Affiliations:** Department of Cognitive Science, Macquarie University, Sydney, NSW, Australia

**Keywords:** mindfulness, organisational citizenship behaviours, implicit bias, decision making, age bias, sunk-cost bias

## Abstract

Mindfulness is becoming increasingly popular in the workplace. This likely relates to a growing body of research linking mindfulness to a range of psychological outcomes such as reduced anxiety, depression and increased subjective wellbeing. However, while mindfulness has received a great deal of attention in clinical research, the evidence for workplace relevant benefits is less established. Additionally, outside of clinical research, mindfulness studies have rarely been replicated. Recent evidence suggests that the cognitive skills cultivated during meditation may be instrumental in reducing biased thinking and increasing prosocial behaviour, but these findings have not been previously tested in a workplace setting. Specifically, mindfulness has been linked to reductions in implicit age bias, sunk-cost decision-making bias and increases in organisational citizenship behaviours (OCB). In two experiments using a workplace and laboratory sample, the present investigation aimed to test the reliability and generalisability of previous findings that a brief mindfulness meditation can reduce age and sunk-cost decision-making biases. To more directly test the potential positive benefits of mindfulness in a workplace setting, this study also investigated the impact of a mindfulness intervention on intention to perform OCB. While meditation significantly increased OCB intent, predictions relating to bias were not supported. Considerations for the degree to which empirical evidence aligns with claims in popular culture, along with implications for the practical uses of mindfulness in the workplace are explored.

## Introduction

Mindfulness programs are now common in organisations with large corporations, such as Google and Intel, using mindfulness as a tool for enhancing employee wellbeing and productivity (Schaufenbuel, 2015; Steward, 2015). Mindfulness can be cultivated through a range of practices. The most well-established technique in western psychology is mindfulness meditation (Good et al., 2016), although there is also evidence that mindfulness can be developed outside of the context of meditation through increased attention to novelty (Langer, 1992, 2014). While definitions of mindfulness vary, in western academic research, it is typically understood as a state of sustained attention to experiences occurring in the present moment (Brown and Ryan, 2003). These experiences are observed as they are, without manipulation, discrimination or resistance. Mindfulness can also be conceived as a trait, such that some people may generally have more capacity to enter mindful states than others (Kabat-Zinn, 2003).

The popularity of mindfulness relates to a growing body of research linking mindfulness to a range of psychological and physiological outcomes. Brief mindfulness interventions have been linked to reductions in stress, negative affect, symptoms of depression, anxiety, chronic pain and increases in subjective wellbeing (Baer, 2003; Khoury et al., 2013; Good et al., 2016; Ngnoumen and Langer, 2016; Sanko et al., 2016). Long-term mindfulness practice is correlated with structural changes in brain areas associated with executive attention and emotion regulation (e.g., Hölzel et al., 2011; Fox et al., 2014). However, despite promising evidence supporting the benefits of mindfulness in laboratory and clinical settings, relatively little is understood about how these benefits might translate to more practical workplace aspects of performance and behaviour (Gallant, 2016). Most research has been conducted in university settings with samples consisting of paid participants or students, potentially limiting the generalisability of findings to corporate working populations.

Evidence suggests that the skills cultivated during meditation may be instrumental in reducing biased thinking and increasing prosocial behaviour (Kiken and Shook, 2011; Hopthrow et al., 2017; Oyler et al., 2021), possibly through an increase in cognitive flexibility (Moore and Malinowski, 2009; Colzato et al., 2016), although this has not yet been tested *via* a randomised and controlled experiment in a workplace setting. Specifically, mindfulness has been linked to increases in organisational citizenship behaviours (OCB; Reb et al., 2015), reductions in implicit age bias (Lueke and Gibson, 2014) and reductions in sunk-cost decision-making bias (Hafenbrack et al., 2014). Responding to a call for greater scientific rigor in investigating the practical benefits of mindfulness meditation (Van Dam et al., 2017), the present investigation aims to explore whether these findings translate to a corporate working population.

### Mindfulness and Bias: A Dual-Process Perspective

Mindfulness practice may reduce biased thinking by influencing how individuals process new information. Dual-process theories of reasoning propose that there are two main ways of processing information: automatic and immediate vs. controlled and explicit (Kahneman, 2011; Evans and Stanovich, 2013). The automatic and immediate style relates to experience-based decision-making, using mental heuristics, and prior automatic associations to guide quick and reflexive decisions that demand minimal cognitive resources. Conversely, the explicit and controlled style requires systematic processing of information in a slow, serial and attentionally demanding manner (Frankish, 2010). As the automatic and immediate style does not require deliberation on all available knowledge, it is associated with more biased decision-making (Evans and Curtis-Holmes, 2005). Conversely, reliant on semantic knowledge rather than implicit assumptions, the explicit and controlled style is associated with less biased decision-making than automatic thinking styles (Evans, 2003). According to dual-process theories, because humans may have a limited cognitive capacity, to conserve cognitive resources, the automatic style may be employed by default, whereas the conscious, effortful, explicit style is likely to be only applied where necessary (Evans and Stanovich, 2013).

Mindfulness may increase prosocial behaviour and reduce biased thinking *via* increasing the use of the explicit and controlled thinking style (Hyland et al., 2015). There are two potential mechanisms by which this may occur: (1) broadening an individual’s awareness of information in the present moment and (2) increasing one’s cognitive capacity.

#### Present Moment Awareness

Mindfulness is the practice of cultivating an un-manipulated perception of the present moment. Accurately perceiving the present moment requires serial analysis of the available information (the controlled and explicit thinking style) rather than automatic assumptions (the automatic and immediate thinking style; Shapiro et al., 2006). A reduced reliance on automatic thinking styles following meditation is supported by research associating mindfulness with an increased ability to respond based on new information rather than habitual responses, this may be associated with an increased openness to new information (Frewen et al., 2008; Ostafin et al., 2012). For example, Ostafin and Kassman (2012) found that university students performed better on insight problem-solving questions (that must be answered using present facts rather than prior associations) following 10-min of meditation compared to a relaxation control. Similarly, a brief mindfulness meditation was found to increase performance on two cognitive tasks that required participants to quickly categorise information using new, non-habitual groupings (e.g., the colour red and the word blue) rather than prior automatic associations (e.g., the colour red and the word red; Wenk-Sormaz, 2005). Together, these findings imply that mindfulness reduces reliance on automatic thinking styles and increases controlled and explicit reasoning.

Present moment awareness also increases consideration of both external and internal cues, enabling greater objectivity in judgements (Hyland et al., 2015). By contrast, reliance on internal cues when processing information (internal encoding), is characteristic of the automatic style of thinking, whereby judgements are made from prior assumptions and heuristics rather than external cues. Internal encoding can be associated with recalling false memories based on prior experiences (Roediger and McDermott, 1995; Dehon et al., 2011). Internal encoding is also correlated with self-perpetuation errors, whereby one maintains a pre-existing belief despite a consistent lack of supporting evidence (Hill et al., 1989). An example of a self-perpetuation error is where an individual may believe an older worker is less effective than a younger worker, despite a lack of evidence indicating inferior work, or after encountering a high-performing older worker. By shifting one’s focus towards a greater range of available information, mindfulness may assist individuals in detecting external cues that correct prior errors in judgement (Herndon, 2008).

#### Increased Cognitive Capacity

Mindfulness also enhances an individual’s ability to engage in controlled and explicit reasoning styles by expanding one’s cognitive capacity. Both brief and long-term mindfulness interventions are linked to improved performance on tasks measuring working memory and sustained attention (e.g., Jha et al., 2007; Chambers et al., 2008; Zeidan et al., 2010). This is supported by the findings that long-term mindfulness practitioners show greater grey matter volume in areas of the brain linked to executive attention, working memory and self-regulation than matched novices (e.g., Froeliger et al., 2012; Fox et al., 2014; Villemure et al., 2015). Longitudinal studies show changes in similar brain regions following 8 weeks of meditation (e.g., Hölzel et al., 2011; Kilpatrick et al., 2011), providing evidence for a causal relationship between mindfulness practice and increased cognitive capacity. Overall, this evidence supports the view that mindfulness enhances aspects of cognitive capacity required to engage in explicit reasoning styles.

### Mindfulness and Positive Social Attitudes

The shift towards deliberative processing during mindfulness may be associated with increased orientation towards external social cues (Herndon, 2008). Greater attention to cues from others may in turn promote a greater ability to empathise and correspondingly, promote more positive social attitudes. Indeed, both trait and state mindfulness have been linked to increases in aspects of empathy, such as perspective taking and feelings of compassion towards others (Shapiro et al., 1998; Block-Lerner et al., 2007; Krasner et al., 2009; Birnie et al., 2010; Boellinghaus et al., 2014). In addition, individuals show increased explicit and implicit positive feelings towards strangers (mediated by an increase in positive feelings towards others) and reduced racial bias following as little as 7-min of meditation (Hutcherson et al., 2008; Stell and Farsides, 2016).

These increased positive feelings towards others may translate to more frequent prosocial behaviours (Good et al., 2016). Trait and state mindfulness have been linked to self-reported real-life helping behaviour and greater cooperation in computer-based economic games (Weng et al., 2013; Cameron and Fredrickson, 2015). Consistent with these findings, Condon et al. (2013) found that individuals were significantly more likely to give up their chair for a disabled confederate following 8 weeks of meditation than following a wait-list control. A moderate effect size (*φ* = 0.36) was found even when a bystander manipulation was introduced whereby other seated confederates displayed no care for the disabled confederate. Together, these findings suggest that mindfulness could be used to promote prosocial behaviour.

### Organisational Citizenship Behaviours

While growing evidence suggests that mindfulness may be used to promote prosocial behaviour, whether these effects transfer to the workplace has received little investigation (Good et al., 2016). In the workplace, prosocial behaviour is often operationalised in terms of OCB. OCBs are behaviours beyond an employee’s contractual obligations that benefit others in the workplace. OCBs include altruism towards one’s colleagues, representing the organisation positively outside of the work context and taking on tasks beyond one’s role (Smith et al., 1983). OCBs are attracting increasing attention in the field of organisational psychology due to links with increased job satisfaction and reduced turnover intention (LePine et al., 2002; Tsai and Wu, 2010). Building on evidence suggesting that mindfulness may promote positive social attitudes and prosocial behaviours, here we investigate whether mindfulness also increases OCBs. Correlational and cross-sectional research provides preliminary support for this, showing a link between mindfulness practices, trait mindfulness and OCBs (Shekari, 2014; Reb et al., 2015; Patel, 2017; Petchsawang and McLean, 2017; Nguyen et al., 2019). However, the relationship between mindfulness and OCBs has not yet been tested in an experimental research design. The first aim of the present study was to fill this gap by empirically testing the effect of mindfulness on intention to engage in OCBs.

### Bias in the Workplace

In addition to potentially encouraging beneficial behaviours, mindfulness may also be protective against common biases. Two forms of bias resulting from using automatic rather than controlled thinking styles that can be extremely costly to organisations are prejudiced implicit attitudes and over-reliance on heuristics in decision-making.

#### Implicit Age Bias

Mindfulness may provide a potential tool for altering implicit attitudes. Implicit attitudes are automatic associations unconsciously held by an individual (Greenwald et al., 2002). Implicit attitudes are an important issue in the workplace due to their significant link to overt behaviour. Indeed, they are consistently more reliable predictors of negative out-group behaviour than self-reported, explicit attitudes (Greenwald et al., 2009). Age bias is a common and concerning form of negative implicit attitude that is common across workplaces (Rupp et al., 2006; Finkelstein et al., 2018). These attitudes can impact workplace decisions related to recruitment, compensation and advancement opportunities (Dennis and Thomas, 2007). In fact, it has been argued that implicit attitudes more strongly predict discriminatory decisions than do explicit attitudes (Rudman and Glick, 2001; Ziegert and Hanges, 2005; Rooth, 2010).

Implicit age biases can be assessed through the Implicit Association Test (IAT), a computer-based behavioural task that measures the strength of automatic associations between concepts in memory (Greenwald et al., 2003). IAT scores can give an indication of both hidden and explicit attitudes towards particular groups (McConnell and Leibold, 2001; Dovidio et al., 2002; Gawronski, 2002). IAT scores have proven resistant to change using interventions attempting to address prejudice in an explicit manner (Malinen and Johnston, 2013).

Lueke and Gibson (2014) measured responses to an IAT task, assessing attitudes related to age, following either a 10-min mindfulness audio recording or a 10-min descriptive control in undergraduate university students. Those in the mindfulness condition showed significantly less implicit age bias on the IAT. As Lueke and Gibson (2014) only tested undergraduate university students, whether this finding translates to a working population is an important question. The second aim of the present study was to assess whether the finding of reduced implicit bias following meditation, could be reproduced in a corporate working population.

#### Decision-Making: The Sunk-Cost Bias

Mindfulness may also reduce the use of biased heuristics in making work-related decisions. One common heuristic is the sunk-cost bias. The sunk-cost bias is the tendency to continue with a project once time, money or resources have been invested, even when evidence that relinquishing the project may provide a better overall outcome is presented (Arkes and Blumer, 1985). This bias relates to placing more weight on past investments or anticipated future regret rather than objectively evaluating present facts during decision-making (Keil et al., 1995; Wong and Kwong, 2007). The sunk-cost bias becomes a problem for organisations when individuals continue investing in failing projects in the hopes that the original investment will be recouped. Thus, the sunk-cost bias can result in elevated losses. For instance, individuals who have invested money in a project expect the project to be more successful than those who have not invested money in the same project (Arkes and Blumer, 1985). Consequently, managers may continue to invest money and resources in failing projects that have received high investment (Parayre, 1995).

Evidence suggests that mindfulness may increase resistance to the sunk-cost bias. Hafenbrack et al. (2014) compared the effects of a 15-min meditation and a 15-min mind-wandering control (where participants were repeated asked to think about whatever came to mind) on resistance to the sunk-cost bias in university students. They found that 78% of those in the mindfulness condition resisted the sunk-cost bias whereas only 44% resisted the bias in the control condition. Hafenbrack et al. (2014) found the same relationship when the experiment was replicated in a paid, online sample. These experiments suggest that both trait and state mindfulness may increase the likelihood of resisting the sunk-cost bias. As discussed above, a feature of mindfulness is increased present-moment focus. It has been shown that focusing more on the present situation rather than past costs or anticipated regret can reduce the sunk-cost bias, perhaps by inviting awareness of new opportunities that may arise from abandoning previous efforts (Wong and Kwong, 2007; Strough et al., 2011). However, these effects have only been shown in first-year university students and a paid online sample, making generalisability unclear. The final aim of the present investigation was to assess whether mindfulness could increase sunk-cost bias resistance in a working population.

### The Current Study

We aimed to explore the relationship between mindfulness and OCB intent. Additionally, we aimed to replicate effects identified by Lueke and Gibson (2014) on implicit age bias and Hafenbrack et al. (2014) on sunk-cost bias resistance in a corporate working sample.

### Hypotheses

We predicted that a mindfulness intervention would increase deliberative reasoning. Specifically, compared to a relaxation control intervention, we hypothesised that a mindfulness intervention would: (1) increase intentions to perform OCBs; (2) reduce implicit age bias; (3) reduce explicit age bias; and (4) increase sunk-cost bias resistance.

## Experiment One

### Method

Experiment one assessed whether a brief mindfulness meditation increased OCB intent and reduced bias, compared to a relaxation control in a working population. This experiment utilised a repeated measures design with participants experiencing both a meditation and control intervention, in counterbalanced order, on two different occasions separated by a week or more. Approval for this research was granted by the Macquarie University Human Ethics Committee. The study was preregistered at https://osf.io/mtgqc. Data and Supplementary Materials for both studies can be found at https://osf.io/y4mju/.

### Participants

Fifty participants working in corporate settings in Sydney, Australia were recruited. Thirty-eight of these were recruited from a large finance corporation *via* flyers distributed within the workplace and emails. Twelve were recruited *via* social media posts (Linkedin and Facebook). Advertisements specified that participants needed to be working at least 8 h a week in a corporate office in Sydney. A $200 gift voucher prize draw was offered as a participation incentive. Three participants were excluded for reporting that they did not pay attention or for not listening during the intervention (listening scale score of 5). In the final sample, ages ranged from 23 to 55 (*M* = 34.32, *SD* = 7.66). Thirteen participants were males and 34 were females. Thirty-seven worked full-time and 10 worked part-time. Thirty participants had practiced meditation in the last 6 months. Of these, 10 practiced regularly (at least once per week).

### Materials

#### Mindfulness Intervention

The mindfulness intervention consisted of a 10-min guided mindfulness meditation audio recording developed by Cropley et al. (2007) and also used by Lueke and Gibson (2014). This recording instructed participants to gently direct their attention to the present moment, specifically, their bodily sensations (breath and heartbeat). Participants were directed to attend to these sensations without control, judgement or resistance. The recording was played digitally through a computer.

#### Relaxation/Control Intervention

The relaxation/control intervention consisted of a 10-min natural history audio recording originally developed by Cropley et al. (2007) and also used by Lueke and Gibson (2014). This recording discussed features of the English countryside in the same voice as the mindfulness intervention. An advantage of an active control condition is that it allows us deduce that the results are related to the active effects of the mindfulness intervention, rather than general effects of participating in an intervention.

#### Demographics Questions

A demographics questionnaire collected information regarding participants age, employment status, hours of work per week, cultural background, gender and previous mindfulness experience.

#### The Mindfulness Attention Awareness Scale (MAAS)

The MAAS measures trait mindfulness, the attribute of being mindful in general life (Brown and Ryan, 2003). The scale consists of 15 self-statements (e.g., ‘*I find it difficult to stay focused on what’s happening in the present*’). Participants rated the extent to which each statement applied to them on a six-point Likert scale, from *1 = almost always* to *6 = almost never*. Scores are summed, with a higher score indicating greater trait mindfulness. The MAAS has previously demonstrated good test–retest reliability (*r* = 0.81), consistent with the scale’s function as a trait measure. Internal consistency has been previously demonstrated as good, Cronbach’s *α* = 0.86 (Brown and Ryan, 2003; Baer et al., 2006). For the current study, Cronbach’s α was 0.91.

#### The Implicit Association Test (IAT)

The IAT is a behavioural, computer-administered measure of implicit attitudes (Greenwald et al., 1998). The present investigation used the same parameters as Lueke and Gibson (2014), measuring attitudes towards old and young age groups. Stimuli for the IAT were images of six old and six young faces and eight positive word (Joy, Pleasure, Happy, Peace, Love, Glorious, Laughter) and eight negative words (Hurt, Nasty, Terrible, Agony, Awful, Horrible, Evil). The IAT was delivered using the EasyIAT Program (Thompson et al., 2016) and was presented in seven blocks, consistent with standard IAT methodologies (see Supplementary Tables S1 and S2; Greenwald et al., 2003).

In the IAT, participants were asked to sort the images and words into categories of ‘good’, ‘bad’, ‘old’, and ‘young’ as quickly as possible. Categories were displayed on the left and right sides of a black screen with the stimuli presented in the middle of the screen. Test blocks displayed one word and one image category on each side of the screen. Pressing the ‘E’ key indicated that an item belonged to the category displayed on the left. Pressing the ‘I’ key indicated that the item belonged to the category on the right. Reaction times on stereotype congruent blocks (e.g., ‘old’ paired with ‘bad’) were compared to reaction times on stereotype incongruent blocks (e.g., ‘old’ paired with ‘good’).

Responses were scored using the D-Scoring algorithm from Greenwald et al. (2003) without error penalties. A higher D-score indicated greater bias towards young over old individuals. The IAT has previously demonstrated satisfactory test–retest reliability (*r* = 0.60) and high internal consistency Cronbach’s *α* = 0.80 (Greenwald et al., 2002).

#### Explicit Bias

The explicit bias question was drawn from the Project Implicit Website and consisted of a single item measuring explicit attitudes towards old and young people (Project Implicit, 2011). Participants were asked to score the statement which best described them on a seven-point scale from 1, *‘I strongly prefer old people to young people’* to 7 *‘I strongly prefer young people to old people’*. Higher scores indicated greater explicit bias towards young people and lower scores indicated greater bias towards old people. A score of 4 indicated no preference for old or young people.

#### Sunk-Cost Resistance Test

The Sunk-Cost Resistance Test consists of two hypothetical scenarios measuring resistance to the sunk-cost bias and used by Hafenbrack et al. (2014). Participants answered ‘yes’ or ‘no’ to each scenario to indicate their decision (see Supplementary Materials: https://osf.io/y4mju/). Correct answers indicated greater sunk-cost resistance. The two scenarios were used as alternate forms, administered after each intervention (mindfulness and control) in counterbalanced order (Arkes and Blumer, 1985).

#### Organisational Citizenship Behaviour Checklist (OCB-C)

The OCB-C is a 24-item self-report questionnaire (Fox et al., 2012). The questionnaire was adapted from the original retrospective format to measure projected OCBs. To achieve this, the original instruction ‘*How often have you done each of the following things on your present job?*’ was changed to ‘*Thinking about your job, in the next few months how compelled do you feel to engage in the following things?*’ Additionally, the structure of the questions was changed to future tense, for example, ‘*Lent a compassionate ear when someone had a work problem*’ became *‘Lend a compassionate ear when someone has a work problem’.* Participants rated the extent to which they felt compelled to engage in each behaviour on a five-point Likert scale, from *1 = not at all* to *5 = a great deal*. Scores were summed with higher values indicating greater intention to perform OCBs. The OCB-C has previously demonstrated high internal consistency, Cronbach’s *α* = 0.89 and satisfactory predictive validity based on co-worker ratings (*r* = 0.29; Fox et al., 2012). The OCB-C was split into two equal alternative forms that were administered after each intervention (mindfulness and control) in counterbalanced order. Cronbach’s *α* was 0.61 and 0.77 for these split versions, demonstrating acceptable reliability.

#### State Mindfulness

The state mindfulness measure, used by Hafenbrack et al. (2014), assessed the extent to which participants felt they were in a state of mindfulness during the interventions. The measure consists of three questions (e.g., ‘*To what extent were you focused on your breathing during the audio recording?*’) answered on a 5-point Likert scale from *1 = Always* to *5 = Never*. The total score was reversed such that a higher score indicated greater state mindfulness.

#### Listening Scale

A single item listening scale was used to assess the extent to which participants were listening during the intervention (‘To what extent where you listening during the recording?’). This item was scored on a five-point Likert scale from *1 = Always* to *5 = Never*.

#### Distraction Scale

A single item distraction scale was used to assess the extent to which participants were distracted during the intervention (‘*Did you do anything else while the recording was playing (e.g., open another window or read something)*?’). This item was scored on a five-point Likert scale from *1 = Always* to *5 = Never*.

#### Social Desirability Scale (SDS-17)

The SDS-17 is a 17-item self-report measure of socially desirability, consisting of self-statements (e.g., ‘*I sometimes litter*’; Stöber, 2001). Participants responded ‘yes’ or ‘no’ to indicate whether each statement applied to them. Summed scores represented each participant’s total, where a higher score indicated greater social desirability. The SDS-17 has previously demonstrated satisfactory to high internal consistency, Cronbach’s *α* = 0.70–0.92 (Blake et al., 2006). For this study, Cronbach’s *α* was 0.64.

### Procedure

Experiment one was implemented as an online study in two parts. In part one participants received an email containing a link to begin the experiment. In the email, participants were reminded that participation was voluntary and instructed to only begin the experiment when they had a laptop or desktop computer, headphones and 30-min of uninterrupted time. Following an auditory sound check (to ensure that sounds could play successfully through the headphones), participants completed the demographic questions and the MAAS. They then listened to either the 10-min mindfulness recording or the 10-min control recording (random allocation). Next, participants completed the IAT. Following this, one Sunk-Cost Resistance Test and OCB-C form were presented in counterbalanced order. Finally, participants completed the State Mindfulness, Listening and Distraction scales, the SDS-17 and the Explicit Bias question. Part one took approximately 30-min to complete.

An email was sent 1 week later requesting participants to complete part two of the experiment. An additional follow-up email was sent after another week had passed to those who had not yet completed part two. Part two followed the same procedure as part one, but omitted the demographics questions, MAAS and SDS-17. Alternate forms of the audio recording, Sunk-Cost Resistance Test and OCB-C were presented. Part two took approximately 20-min to complete.

## Experiment One Results

### Manipulation Check

To test the effectiveness of the meditation intervention in inducing mindfulness, we compared State Mindfulness scores between the experimental conditions. State mindfulness was significantly higher following the mindfulness intervention (*M* = 10.98, *SD* = 2.18) than following the control intervention (*M* = 6.81, *SD* = 2.16), *F*(1,46) = 113.72, *p* < 0.001, *η*^2^ = 0.71, suggesting that the meditation intervention was leading participants to experience subjective changes in their psychological state. Additionally, participants reported spending significantly more time listening during the mindfulness intervention (*M* = 1.89, *SD* = 0.84) than during the control intervention (*M* = 2.66, *SD* = 0.89), *F*(1,46) = 21.71, *p* < 0.001, *η*^2^ = 0.32. Participants reported no difference in distraction during the mindfulness (*M* = 2.81, *SD* = 1.62) and control (*M* = 3.09, *SD* = 1.54) intervention recordings (*F*(1,46) = 0.39, *p* = 0.535, *η*^2^ = 0.01). Descriptive statistics for all outcome variables are shown in Table 1.

**Table 1:** Descriptive statistics for dependent variables by condition.

Measure	Condition	
	Mindfulness	Control
OCB intent	26.34 (5.51)	24.75 (4.83)
Implicit bias	0.38 (0.35)	0.37 (0.29)
Explicit bias	3.74 (0.99)	3.55 (1.23)
Sunk-cost resistance	0.38 (0.49)	0.43 (0.50)

Scores are mean values with SDs in parentheses.

### Analysis Strategy

For each dependent variable, we investigated the difference between the relaxation and mindfulness conditions, controlling for Trait Mindfulness. For OCB Intent and Explicit Bias, we additionally controlled for Social Desirability (using the SDS-17) due to the susceptibility of these scales to demand characteristics.

### Organisational Citizenship Behaviour Intent

Consistent with Hypothesis One, a repeated measures ANCOVA with intervention type as a within subjects factor, and Social Desirability and Trait Mindfulness as covariates, revealed that OCB was significantly higher in the mindfulness condition (*M* = 26.78, *SD* = 5.13) than the control condition (*M* = 24.42, *SD* = 4.56), *F*(1,44) = 5.48, *p* = 0.024, *η*^2^ = 0.11.

### IAT (Implicit Age Bias)

Contrary to Hypothesis Two, a repeated measures ANCOVA, controlling for Trait Mindfulness, revealed no significant difference in Implicit Age Bias between the mindfulness (*M* = 0.38, *SD* = 0.35) and control interventions (*M* = 0.37, *SD* = 0.29), *F*(1,45) = 0.96, *p* = 0.333, *η*^2^ = 0.02.

### Explicit Age Bias

Contrary to Hypothesis Three, a repeated measures ANCOVA, controlling for Social Desirability and Trait Mindfulness, revealed no significant difference in Explicit Age Bias between the mindfulness (*M* = 3.74, *SD* = 0.99) and control interventions (*M* = 3.55, *SD* = 1.23), *F*(1,44) = 0.00, *p* = 0.981, *η*^2^ = 0.00.

### Sunk-Cost Bias

Contrary to Hypothesis Four, a binary logistic regression analysis, controlling for Trait Mindfulness, showed no significant difference in Sunk-Cost Resistance between the mindfulness (*M* = 0.38, *SD* = 0.49) and control interventions (*M* = 0.43, *SD* = 0.50), *Wald χ*^2^ (1, *N* = 94) = 1.01, *p* = 0.315, *OR* = 1.19.

### Exploratory Analyses

Table 2 shows correlations between measures in Experiment one. Of note, were patterns of association between Trait Mindfulness, State Mindfulness, Social Desirability and key outcome measures. There was an association between State Mindfulness and OCB Intent following the mindfulness intervention (*r* = 0.41, *p* = 0.005), such that individuals who experienced greater levels of state mindfulness had greater positive intentions for prosocial work behaviours. Higher levels of Trait Mindfulness was associated with greater State Mindfulness in the control condition (*r* = 0.31, *p* = 0.039), but not in the mindfulness condition. Surprisingly, Trait Mindfulness was also associated with increased implicit bias in the mindfulness condition (*r* = 0.313, *p* = 0.032). Social desirability was negatively associated with Trait Mindfulness (*r* = −0.33, *p* = 0.025) and also negatively correlated with State Mindfulness following both the mindfulness (*r* = −0.41, *p* = 0.005) and control (*r* = −0.29, *p* = 0.047) interventions. Together these findings indicate that increased levels of mindfulness are associated with reduced tendency to give socially desirable responses.

**Table 2:** Associations between measures in Study 1.

	MAAS	SDS	SM med	SM con	OCB med	OCB con	IAT med	IAT con	Explicit med	Explicit con	Sunk-costs med
SDS	−0.326^*^										
SM med	0.178	−0.406^**^									
SM con	0.302^*^	−0.291^*^	0.239								
OCB med	−0.071	−0.249	0.407^**^	0.080							
OCB con	0.174	0.006	0.030	0.014	0.064						
IAT med	0.313^*^	−0.144	0.083	−0.116	0.086	0.015					
IAT con	0.182	0.116	0.078	−0.029	−0.028	0.046	0.361^*^				
Explicit med	0.173	−0.061	0.169	0.251	0.028	0.282	−0.179	−0.223			
Explicit con	0.175	−0.092	0.191	0.220	−0.025	0.054	−0.021	−0.106	0.727^**^		
Sunk-costs med	−0.005	−0.044	−0.094	0.009	−0.210	−0.333^*^	−0.155	0.156	−0.018	0.073	
Sunk-costs con	0.042	−0.302^*^	0.008	−0.104	0.120	0.082	0.068	−0.092	0.005	−0.144	−0.147

MAAS, mindful attention awareness scale; SDS, social desirability scale; SM, state mindfulness scale; OCB, organisational citizenship behaviour; IAT, implicit attribution task; Med, meditation condition; and Con, control condition.

* Correlation is significant at the 0.05 level.

** Correlation is significant at the 0.01 level (2-tailed).

## Experiment One Discussion

The hypothesis that the mindfulness intervention would lead to an increase in OCB intent was supported in experiment one. However, the hypotheses relating to cognitive biases (i.e., implicit age bias and sunk-cost resistance) were not supported. The lack of findings related to biases are surprising, particularly considering that this study closely followed the parameters of Lueke and Gibson (2014), who found a clear effect of mindfulness on implicit biases. Additionally, increases in sunk-cost bias resistance were previously found in two experiments by Hafenbrack et al. (2014) following a similar mindfulness intervention. The present study used identical stimuli, interventions and measures to Lueke and Gibson, but differed slightly in design: Lueke and Gibson tested student participants in a between-subject design in a laboratory setting, whereas experiment one tested working adults in a repeated measures design that was implemented as an online study.

In light of these findings, experiment two aimed to directly replicate the design of Lueke and Gibson (2014) in order to confirm that the findings regarding cognitive biases are indeed replicable. Experiment two used an identical between-subject, laboratory design and recruited a sample from the same population (students) as Lueke and Gibson.

## Experiment Two

### Design and Procedure

Experiment two utilised a between-subject laboratory design. Participants were randomly allocated to either the mindfulness or control condition. Following written informed consent, participants were directed to individual computer booths. The experiment then followed the same procedure as part one of experiment one, except that full versions of the OCB-C and Sunk-Cost Resistance Tests were administered (rather than the split-half versions used in experiment one). Due to the controlled setting, the distraction scale was removed. The entire procedure took approximately 30-min to complete. Cronbach’s *α* was 0.85 for the MAAS, 0.70 for the OCB-C and 0.62 for the SDS-17.

### Participants

One hundred and nineteen undergraduate psychology students participated for course credit. Ages ranged from 17 to 53 (*M* = 21.66, *SD* = 7.54). Twenty-eight were male and 91 were female. Nine worked full-time, 44 worked part-time, 65 worked casually and one participant was unemployed. Seventy-two had practiced meditation in the last 6 months. Of these, six practiced regularly (at least once per week).

## Experiment Two Results

### Preliminary Data Analysis

Baseline Demographic and Trait Mindfulness scores were compared to check randomisation between groups (mindfulness or control). Groups did not differ in age [*F*(1,117) = 2.42, *p* = 0.123, *η*^2^ = 0.02], hours of work per week [*F*(1,117) = 0.05, *p* = 0.824, *η*^2^ = 0.00], Trait Mindfulness [*F*(1,117) = 0.06, *p* = 0.808, *η*^2^ = 0.00] or prior mindfulness experience [*F*(1,117) = 0.50, *p* = 0.478, *η*^2^ = 0.00]. Descriptive statistics for all outcome variables are shown in Table 3.

**Table 3:** Descriptive statistics for dependent variables by condition.

Measure	Condition	
	Mindfulness	Control
OCB intent	73.67 (8.99)	70.79 (9.67)
Implicit bias	0.36 (0.25)	0.42 (0.33)
Explicit bias	3.57 (1.16)	3.55 (1.37)
Sunk-cost resistance	0.59 (0.67)	0.55 (0.60)

Scores are mean values with SDs in parentheses. OCB and sunk-cost scores are increased relative to Experiment 1 as full versions of these measures were used.

### Manipulation Check

State Mindfulness was significantly higher following the mindfulness intervention (*M* = 9.05, *SD* = 1.91) compared to the control intervention (*M* = 5.43, *SD* = 2.08), *F*(1,117) = 97.50, *p* < 0.001, *η*^2^ = 0.00. Additionally, participants in the mindfulness condition reported spending significantly more time listening (*M* = 1.64, *SD* = 0.731) than those in the control intervention (*M* = 3.00, *SD* = 0.838), *F*(1,117) = 89.37, *p* < 0.001, *η*^2^ = 0.43.

### Organisational Citizenship Behaviour Intent

One participant was excluded from all OCB analyses because they did not meet the inclusion criteria of working at least 8 h per week, and a further participant was excluded for reporting that they did not understand the OCB questions. Consistent with Hypothesis One, an ANCOVA controlling for Social Desirability and Trait Mindfulness revealed a significant difference in OCB Intent scores between the mindfulness and control interventions, *F*(1,113) = 4.72, *p* = 0.032, *η*^2^ = 0.04. This relationship was such that those in the mindfulness condition showed significantly higher OCB Intent than those in the control condition.

### Implicit Age Bias

Due to a data collection error, six participants failed to complete the IAT and were excluded from the following analyses. Contrary to Hypothesis Two, an ANCOVA, controlling for Trait Mindfulness, revealed no significant difference in Implicit Age Bias between the mindfulness and control interventions, *F*(1,110) = 1.07, *p* = 0.303, *η*^2^ = 0.01.

### Explicit Age Bias

Contrary to Hypothesis Three, an ANCOVA controlling for Social Desirability and Trait mindfulness revealed no significant difference in Explicit Age Bias between the mindfulness and control interventions, *F*(1,115) = 0.01, *p* = 0.970, *η*^2^ = 0.00.

### Sunk-Cost Bias

Contrary to Hypothesis Four, a multinomial logistic regression analysis controlling for Trait Mindfulness showed no significant difference in Sunk-Cost Resistance scores between the mindfulness or control interventions, *χ*^2^ (2, *N* = 119) = 1.10, *p* = 0.577, *OR* = 1.14.

### Exploratory Analysis

Table 4 shows correlations between measures in Experiment Two. State Mindfulness was again positively associated with OCB Intent (*r* = 0.18, *p* = 0.046), indicating that individuals who experienced more mindfulness states were more likely to intend prosocial work behaviours. Social Desirability was negatively associated with both Trait Mindfulness (*r* = 0.30, *p* = 0.001) and OCB Intent (*r* = −0.25, *p* = 0.002), indicating that more mindful and prosocial individuals were less likely to answer in an artificial manner. Finally, there was a positive association between Trait Mindfulness and Explicit Bias (*r* = 0.22, *p* = 0.018). However, total scores for Explicit Bias indicated very little bias overall (mean scores for both conditions were <4, indicating a slight preference for older people rather than any stereotypical age bias).

**Table 4:** Associations between measures in Study 2.

	MAAS	SDS	SM	OCB	IAT	Explicit
SDS	−0.301^**^					
SM	−0.036	0.007				
OCB	−0.028	−0.284^**^	0.184^*^			
IAT	−0.027	0.002	−0.070	0.070		
Explicit	0.216^*^	−0.056	−0.005	0.166	−0.030	
Sunk-costs	0.003	0.043	−0.058	0.075	0.088	0.040

MAAS, mindful attention awareness scale; SDS, social desirability scale; SM, state mindfulness scale; OCB, organisational citizenship behaviour; and IAT, implicit attribution task.

* Correlation is significant at the 0.05 level.

** Correlation is significant at the 0.01 level (2-tailed).

## Experiment Two Discussion

The results for experiment two mirror those identified in experiment one. Experiment two again failed to replicate age and sunk-cost bias findings by Lueke and Gibson (2014) and Hafenbrack et al. (2014). Experiment two did replicate findings relating to OCB intent, supporting the reliability of findings from experiment one.

## General Discussion

Across two experiments, using a corporate working sample and a university student sample, the present investigation sought to extend research showing positive social impacts of mindfulness by investigating the impact of mindfulness on OCB intent. Additionally, the present study aimed to assess whether previous findings that a brief mindfulness intervention reduced implicit age bias and increased resistance to the sunk-cost bias could be replicated. Predictions regarding OCB intent were supported by both experiments. However, predictions relating to reductions in bias were not supported by either experiment one or two.

### Organisational Citizenship Behaviours

The first prediction, that mindfulness meditation would lead to greater intention to perform OCBs than a relaxation control intervention was supported in both experiments. These findings are consistent with previous correlational research by Patel (2017), Reb et al. (2015) and Shekari (2014) that showed a link between mindfulness practice and OCBs. Furthermore, these findings imply that prosocial behaviours following mindfulness meditation observed by Condon et al. (2013) are likely to transfer to workplace contexts. The present findings provide preliminary evidence for a linear relationship between mindfulness and OCBs. Additionally, exploratory correlational analyses revealed a positive relationship between state mindfulness and OCB intent in both experiments. Individuals who reported that they were mindful during the intervention were more likely to report prosocial workplace intentions.

While the present findings represent an important step forward in our understanding of the nature of the relationship between mindfulness and OCB intentions, it is important to highlight that the observed effect sizes were small. This may be due to the format of the OCB scale, which required participants to imagine how likely they were to perform a list of OCBs over the next few weeks. The abstract nature of this task may have reduced participant’s ability to respond with precision, reducing the sensitivity of the measure. Future research should investigate this by analysing the impact of mindfulness on OCBs more directly with measures of overt behaviour. This would also help to determine whether self-reported findings translate to actual behaviour or simply reflect positive intentions susceptible to social desirability. The small effect sizes may also reflect the brevity of the mindfulness intervention (only 10-min). An important question moving forward is whether a longer intervention would produce larger effects.

### Implicit Age Bias

The second prediction, that mindfulness would lead to greater reductions in implicit age bias than a relaxation control, was not supported in either the workplace or university samples. This finding is surprising given that this hypothesis aimed to replicate significant findings from a study by Lueke and Gibson (2014). Both of the current experiments used identical measures and intervention audio recordings to the original study. Additionally, Experiment Two replicated the exact procedure used by Lueke and Gibson (2014), including the setting (individual computer labs) and an equivalent sample (first-year university students). Advice was sought from the original researchers to ensure an accurate replication of the original procedure. These considerations reduce the likelihood that the failure to replicate Lueke and Gibson’s findings can be attributed to methodological differences.

One potential explanation for these inconsistent results is that Lueke and Gibson (2014) original findings are not robust to deviations in sample characteristics. Original findings, which assessed first-year university students in the United States of America, may not transfer to an Australian corporate working population (Experiment One). Additionally, cultural differences between American and Australian university students may have led to different experimental effects observed in experiment two. As such, the present null results imply that, brief mindfulness interventions cannot be used to reliably impact implicit age bias.

### Explicit Age Bias

The third prediction, that mindfulness would lead to greater reductions in explicit age bias than a relaxation control, was also unsupported. In fact, scores on this measure indicated no overall preference for either old or young people. This finding is less surprising than the implicit bias results. A key reason for biases being an issue in the workplace is that people are often unaware of biases they hold. This has previously been demonstrated by studies showing differences between implicit bias scores (on the IAT) and explicit self-reported ratings (Malinen and Johnston, 2013). The high face validity of the scale likely led participants to present themselves as not possessing biases.

### Sunk-Cost Bias

The final prediction, that mindfulness meditation would lead to greater resistance to the sunk-cost bias than a relaxation control, was not supported in either experiment. This finding is unexpected given that Hafenbrack et al. (2014) previously observed increases in sunk-cost bias resistance following mindfulness meditation. This inconsistency may be due to methodological differences between studies. Although both studies utilised a similar mindfulness intervention, the present intervention was 10-min in duration, whereas Hafenbrack et al. (2014) intervention was 15-min long. Thus, the present lack of significant effects may suggest that 10-min of mindfulness meditation is not sufficient to reliably impact sunk-cost bias resistance.

An alternative explanation for these unexpected findings relates to differences between the comparison conditions used in the original and present experiments. The present experiments compared a mindfulness audio recording to a relaxation control recording (consistent with Lueke and Gibson, 2014). Conversely, Hafenbrack et al. (2014) compared their mindfulness intervention to a mind-wandering audio induction. While mindfulness involves active attention to features of the present moment, mind-wandering involves passively letting the mind drift towards the past and future. As such, mind-wandering is usually conceptualised as involving the opposite pattern of mental activity to mindfulness (Killingsworth and Gilbert, 2010). This increased distinctiveness between the two conditions in Hafenbrack et al. (2014) experiment may account for the different findings.

### Exploratory

The exploratory correlational analyses revealed a negative association between State Mindfulness and Social Desirability in both experiments. In its essence, Social Desirability is responding in ways that present oneself in a more favourable light. This tendency to present ourselves in favourable ways is often driven by largely unconscious processes. Mindfulness increases one’s deliberation on the present moment, which may in turn make individuals more aware of inaccurate self-attributions. This heightened awareness may increase an individual’s tendency to respond objectively rather than based on unconscious emotional triggers such as social presentation (Ruedy and Schweitzer, 2010). Indeed, previous research has found links between mindfulness and increased ethical behaviour. For instance, individuals high in trait mindfulness are less likely to cheat on anagram tasks (Ruedy and Schweitzer, 2010). Additionally, Shapiro et al. (2012) found an increase in ethical decision-making following an 8-week meditation course. While conclusions about causation cannot be made from the present correlational data, the present findings support the link between mindfulness and ethical behaviour.

### Future Directions

Meditation research in general has been criticised for methodological inconsistencies and low reliability (Sedlmeier et al., 2012). These issues are particularly pertinent in the context of strong public and media interest in the applications of meditation for wellbeing and productivity (Van Dam et al., 2017). Accordingly, it is important to be cautious in making claims based on these results.

The present two experiments showed an influence of mindfulness on OCB intent. These findings support a link between mindfulness and increased attention to one’s impact on others. This may relate to increased deliberate attention to external cues, particularly socially relevant cues. This result is compatible with Sedlmeier et al. (2012) meta-analytic findings showing that the strongest effects in the mindfulness literature related to social attitudes. Conversely, these results suggest that short doses of mindfulness meditation may not be practically useful in changing deep seated implicit reasoning capacities, such as biases. As mindfulness may have more robust impacts on social attitudes, investigations within this domain may yield more practically useful results. Future studies could assess whether the present self-reported attitude change following mindfulness meditation translates into positive workplace behaviour. This could be done, for example, through indirect measures of extra-curricular work activities.

One limitation of the study is that participants differed in prior meditation experience, which may have influenced results. In fact, more than half the participants reported practicing meditation within the last 6 months in both studies. As meditation has become a popular activity, a challenge for research investigating mindfulness in the general population is finding truly naïve samples. Future research would benefit from recruiting carefully pre-screened participants based on lack of prior meditation experience.

Additionally, one important unresolved methodological issue is determining the appropriate duration of mindfulness interventions. The 10-min intervention here had no impact on sunk-cost resistance, whereas 15-min interventions have repeatedly been reported to influence this measure. In general, Western mindfulness interventions vary from single sessions of five to 30-min duration through to an extended period of sessions varying in length. With this wide variation in intervention lengths, the ‘dose–response’ of mindfulness is unclear. For instance, it is unclear whether there is a linear relationship between the amount of mindfulness meditation and the strength of the effects, or whether a plateau effect occurs after a certain amount of meditation. Future research should include meditation interventions of varying length to explore the effect of dose on outcomes.

### Conclusion

In two preregistered studies, we showed that mindfulness intervention led to a marked increase in organisational citizenship intentions. However, we failed to replicate previous findings showing that a brief mindfulness meditation would reduce implicit age bias or sunk-cost bias. These results provide preliminary evidence for a causal relationship between mindfulness practices and prosocial workplace attitudes, although, the degree to which improved attitudes may translate to changes in behaviour is not yet known.

## Data Availability Statement

Data and Supplementary Materials for both experiments can be found at: https://osf.io/y4mju/.

## Ethics Statement

The studies involving human participants were reviewed and approved by Macquarie University Ethics Committee. The patients/participants provided their written informed consent to participate in this study.

## Author Contributions

EW and VP designed the project and analysed the data. EW collected the data and drafted the manuscript. VP reviewed the manuscript. All authors contributed to the article and approved the submitted version.

## Conflict of Interest

The authors declare that the research was conducted in the absence of any commercial or financial relationships that could be construed as a potential conflict of interest.

## Publisher’s Note

All claims expressed in this article are solely those of the authors and do not necessarily represent those of their affiliated organizations, or those of the publisher, the editors and the reviewers. Any product that may be evaluated in this article, or claim that may be made by its manufacturer, is not guaranteed or endorsed by the publisher.
